# The Role of Emergent Brain CT Scans in the Management of Epilepsy Patients Presenting With Breakthrough Seizures

**DOI:** 10.7759/cureus.110068

**Published:** 2026-06-01

**Authors:** Brian Yarahmadi, Benjamin Yi, Akmal Shahzad, Alireza Yarahmadi

**Affiliations:** 1 Neurology, St. George's University School of Medicine, St. George's, GRD; 2 Internal Medicine, MercyOne North Iowa, Mason City, USA; 3 Surgery, St. George's University School of Medicine, St. George's, GRD; 4 Neurology, MercyOne North Iowa, Mason City, USA

**Keywords:** breakthrough, ct, emergency, epilepsy, neuroimaging, neurology, seizures

## Abstract

Background and purpose

Approximately 1-3% of global emergency department (ED) visits are for acute seizures. The role of neuroimaging with non-contrast computed tomography (CT) has been well described for first-time unprovoked seizures. However, current guidelines provide limited or non-specific recommendations for the utility of routine neuroimaging in patients with a known history of epilepsy presenting with breakthrough seizures. This study evaluated the clinical utility of brain CT imaging in adults with a previously confirmed diagnosis of epilepsy who presented to the ED with breakthrough seizures.

Methods

We conducted a retrospective review of electronic medical records from 2019 to 2023. Seizure-specific diagnostic codes were used to identify patients who presented to the ED during the study period. Patients meeting all inclusion criteria were selected for review of CT imaging results and clinical management.

Results

A total of 207 seizure-related ED encounters were identified. After applying inclusion and exclusion criteria, 121 encounters involving adults with known epilepsy presenting with breakthrough seizures who underwent emergent non-contrast head CT were included in the final analysis. Indications for imaging included falls (47, 38.8%), altered mental status (24, 19.8%), lethargy (19, 15.7%), recurrent seizures (20, 16.5%), and headache (11, 9.09%). Among those imaged, CT findings were normal in 92 (76.0%), showed prior stroke in 11 (9.1%), postoperative changes in 9 (7.4%), small-vessel disease in 5 (4.1%), remote trauma in 2 (1.7%), and ambiguous findings in 2 (1.7%). No CT findings represented new pathology compared with prior imaging, and none of these findings altered acute clinical management.

Conclusions

The diagnostic yield of emergent brain CT in epilepsy patients with breakthrough seizures is low. Given the financial burden, exposure to radiation, and limited clinical impact, future studies should focus on developing evidence-based imaging guidelines that minimize unnecessary CT use while maintaining patient safety.

## Introduction

Emergency department (ED) evaluation of patients presenting with seizures, particularly those with known epilepsy experiencing breakthrough events, remains a significant clinical challenge because of the broad differential diagnosis (e.g., structural, toxic, metabolic, infectious etiologies) and the need to rapidly identify potentially life-threatening intracranial pathology [1]. Acute seizures account for approximately 1-3% of ED visits worldwide and necessitate a structured approach to diagnosis and management, aiming to distinguish benign seizure activity from underlying structural lesions such as hemorrhage, infarcts, or mass lesions, which may warrant immediate intervention [2,3]. Non-contrast computed tomography (CT) of the brain is widely available and often used as the first-line imaging study in these cases. It is particularly useful for detecting acute intracranial hemorrhage, mass effect, or signs of trauma that could alter immediate management. For instance, evidence suggests that emergent CT findings can change acute management in a subset of patients with first-time seizures [1,4].

Despite this, guidelines and evidence on the use of emergent CT in patients with known epilepsy presenting with breakthrough seizures remain inconsistent. The American Academy of Neurology’s practice parameters underscore emergent neuroimaging for first-time unprovoked seizures but note insufficient evidence to support routine emergent imaging specifically for breakthrough seizures in established epileptic patients [5]. Moreover, CT utilization patterns vary widely in practice, and recent observational data indicate that head CT scans often yield no acute pathology influencing management in patients with recurrent seizures, particularly when there are no clinical red flags such as persistent focal deficits, trauma, or altered mental status [5,6].

Consequently, there is a critical need to refine clinical decision-making frameworks that balance the potential benefits of rapid identification of emergent pathology with the known risks and limitations of routine CT use, including radiation exposure, cost, and low diagnostic yield in certain clinical contexts [7,8]. This study aims to evaluate the efficacy of emergent brain CT in the ED specifically for epilepsy patients presenting with breakthrough seizures, focusing on its impact on acute management decisions rather than broader diagnostic or longitudinal care pathways.

## Materials and methods

**Figure 1:**
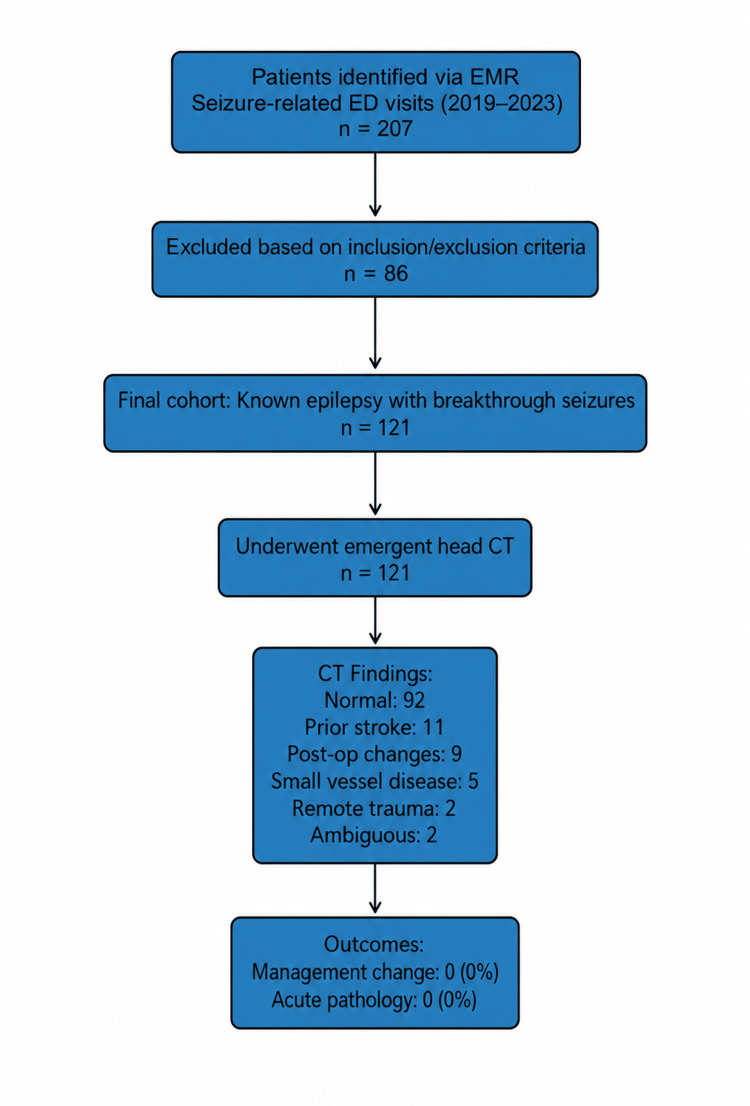
Cohort Selection Flowchart

The study was conducted at a single community-based hospital ED in the United States, which manages a high volume of adult seizure presentations annually and has 24-hour access to brain CT imaging and neurology consultation. Adult patients aged 18-60 years with a documented diagnosis of epilepsy who presented to the ED with a breakthrough seizure during 2019-2023 were identified through electronic medical record (EMR) review using seizure-specific diagnostic codes and ED encounter data. Specific inclusion and exclusion criteria are listed in Table 1. A cohort flowchart illustrating the patient selection process is shown in Figure 1.

**Table 1:** Inclusion and Exclusion Criteria

Category	Criteria
Inclusion Criteria	
	Age 18–60 years
	Established diagnosis of epilepsy
	On antiepileptic medication
	Breakthrough seizure presentation
	Emergent CT obtained
Exclusion Criteria	
	First-time or new-onset seizure
	Not on or noncompliant with antiepileptics
	Pregnancy
	History of status epilepticus or nonepileptic seizure
	Incomplete medical records

Data was extracted retrospectively from the EMR using a standardized data abstraction form. Collected variables included patient demographics, epilepsy history, clinical presentation, whether head CT imaging was obtained, CT findings, and ED management decisions. After primary data collection was complete, patients were grouped based on their CT findings and seizure type for statistical analysis. All data was de-identified at this step. Data analysis was then performed using R software (version 4.4.2; R Foundation for Statistical Computing, Vienna, Austria).

The primary outcome measured was the proportion of emergent head CT scans that resulted in a change in acute clinical management, which, for the purposes of this study, was defined as neurosurgical consultation, initiation of a new antiseizure medication, or additional diagnostic testing directly attributable to CT findings. The secondary outcome was the frequency of clinically significant CT abnormalities, defined as acute intracranial findings caused by or as a consequence of the breakthrough seizure.

## Results

A total of 207 patients were identified from the EMR using seizure-specific diagnostic codes; 121 (58.5%) met the inclusion and exclusion criteria outlined in Table 1 and underwent emergent non-contrast head CT. The specific reasons for obtaining emergent imaging are listed in Table 2.

**Table 2:** Rationale for Obtaining Emergent Head CT

Rationale	n	%
Recent fall	47	38.9
Altered mental status	24	19.8
Recurrent seizures	20	16.5
Lethargy	19	15.7
Headache	11	9.09

To evaluate the primary outcome of this study, which was the proportion of emergent head CT scans that altered clinical management, we performed further retrospective chart review by analyzing ED encounter data. Primary outcome analysis revealed there were no changes in acute clinical management that could be attributed to the findings on emergent CT scans (0/121, 0%, 95% CI 0-2.5%).

**Figure 2:**
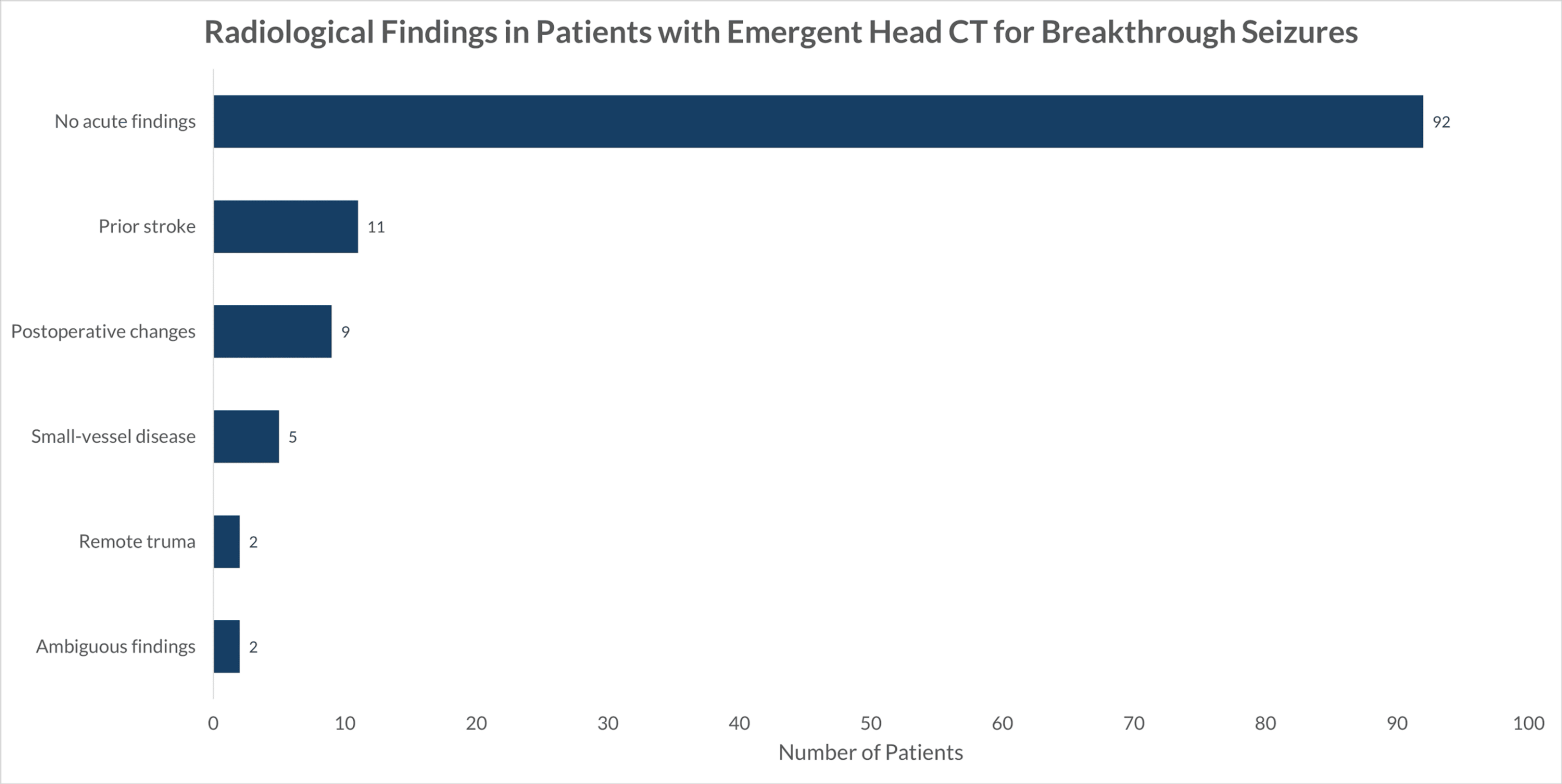
Among 121 patients who underwent emergent head CT for evaluation of breakthrough seizures, the majority of scans were interpreted as no acute findings (n = 92). Abnormal findings primarily reflected chronic or pre-existing pathology, including prior cerebrovascular infarction (n = 11), postoperative changes (n = 9), small-vessel ischemic disease (n = 5), and remote traumatic injury (n = 2). A small number of studies demonstrated nonspecific or ambiguous findings (n = 2). No acute intracranial pathology directly attributable to the seizure event was identified.

We continued analyzing CT reports to assess the secondary outcome of this study, which was the frequency of acute intracranial abnormalities identified on head CT that could be attributed to the breakthrough seizures. All 121 scans demonstrated no clinically significant acute intracranial pathology that could be attributable to the seizure (0/121; 0%, 95% CI 0-2.5%). Despite this, we decided to further categorize patients based on their radiological findings. The most frequently reported finding was a head CT with no acute findings (92/121, 76%). Additional radiological findings and the number of patients per group are reported in Figure 2. Given the absence of clinically significant CT findings and CT-attributable management changes, comparative analyses for predictors of these outcomes were not performed.

## Discussion

In this study of patients with known epilepsy presenting to the ED with breakthrough seizures, non-contrast head CT demonstrated no acute abnormalities beyond baseline findings, and no CT imaging result led to a change in the patient’s emergency or epilepsy management. These findings are consistent with existing evidence that routine CT imaging in patients with previously diagnosed epilepsy has low diagnostic and therapeutic yield [1,5].

Our results, similar to other ED-based studies, demonstrated that CT neuroimaging in patients with established epilepsy and typical breakthrough seizures rarely identifies clinically actionable pathology [5]. This is in contrast to first-time seizure presentations, where CT may have clinical relevance in detecting hemorrhage, mass lesions, or other acute intracranial processes that can cause seizures [9]. Patients with known epilepsy generally have a stable underlying diagnosis and prior neuroimaging. In this context, CT imaging appears to function primarily as a rule-out tool rather than a modality that meaningfully informs treatment decisions.

Importantly, none of the CT scan findings in our cohort revealed new pathology or findings that changed the course of the patient's treatment, including initiation or change in antiseizure medications, escalation of care, neurosurgical consultation, or hospital admission that was specifically related to a CT finding. This suggests that CT imaging did not provide any additive value beyond review of baseline neuroimaging. From a clinical standpoint, our findings support a transition to a selective imaging strategy that would only indicate CT use in specific high-risk patients and not as a routine imaging tool in this patient population.

In the absence of neuroimaging findings that changed the course of patient treatment, patient resource and safety implications in the use of routine CT should be considered. Emergent CT imaging, especially when overutilized, contributes to increased exposure to ionizing radiation, which has been linked in epidemiologic studies to elevated lifetime cancer risk, and places additional financial burden on healthcare systems by increasing costly imaging without clear clinical benefit. Both of these adverse outcomes have been shown to have negative effects on patient care [7,8].

The findings of this study may have potential implications for ED clinical management and imaging decision-making by ED providers. In patients with known epilepsy presenting with a breakthrough seizure who have a normal neurologic exam, return to baseline mental status, and no concern for trauma, infection, or other secondary causes, emergent CT imaging appears to offer limited clinical benefit. Management of these patients should instead focus on identifying likely causes of breakthrough seizures such as medication nonadherence, metabolic derangements, concurrent illness, and factors far more likely to influence immediate treatment.

## Conclusions

In our study cohort, among patients with known epilepsy presenting to the ED with breakthrough seizures, head CT demonstrated no acute abnormalities and did not alter clinical management. These findings support a selective, clinically driven approach to neuroimaging rather than routine CT use in this population. Future prospective studies should focus on establishing evidence-based guidelines and criteria to safely reduce unnecessary imaging while preserving patient safety.
